# Studies on Pitting Corrosion of Al-Cu-Li Alloys Part I: Effect of Li Addition by Microstructural, Electrochemical, In-situ, and Pit Depth Analysis

**DOI:** 10.3390/ma12101600

**Published:** 2019-05-16

**Authors:** Xiaowei Lei, Alireza Saatchi, Elmira Ghanbari, Runze Dang, Wenzhe Li, Nan Wang, Digby D. Macdonald

**Affiliations:** 1 MOE Key Laboratory of Materials Physics and Chemistry under Extraordinary Conditions, School of Natural and Applied Sciences, Northwestern Polytechnical University, Xi’an 710072, China; xiaowei_lei@nwpu.edu.cn (X.L.); dangrunze@mail.nwpu.edu.cn (R.D.); lwz913157048@163.com (W.L.); 2 Department of Materials Science and Engineering, University of California at Berkeley, Berkeley, CA 94720, USA; alireza.saatchi@berkeley.edu (A.S.); elmira.ghanbari@berkeley.edu (E.G.)

**Keywords:** Al-Cu-Li alloys, microstructure, pitting corrosion, polarization, TEM

## Abstract

To analyze the effect of lithium and microstructure on the pitting corrosion behavior of aluminum alloys, three types of aluminum alloys were studied via scanning electron microscopy, transmission electron microscopy, electrochemical polarization, and by immersion tests coupled with in-situ observation of pitting and statistical analysis of pit depths measured by surface profilometry. It was found that, with increasing lithium content, the resistance to pitting corrosion was enhanced and the passive range was enlarged. In-situ observation revealed that the development of pitting corrosion exhibited three stages, including an initial slow nucleation stage (Stage I), a fast development stage (Stage II), and a stabilized growth stage (Stage III). Higher lithium content contributed to shorter time periods of Stages I and II, resulting in faster pitting evolution and a higher number of pits. However, the pits were generally shallower for the specimen with the highest lithium content, which is in agreement with the results of the electrochemical analysis.

## 1. Introduction

The primary driving force for research and development on aviation aluminum alloys is to reduce the weight of aircraft [1,2,3,4]. Owing to the low density, high strength, and excellent fatigue crack resistance, Al-Cu-Li alloys have received increasing attention during the last two decades [5,6,7,8,9,10]. Being the lightest metallic element in the nature, lithium contributes to 6% increase in elastic modulus and 3% decrease in weight for aluminum alloys by adding 1 wt.% of lithium [2,3]. However, as lithium is an active alkali metal, adding lithium may have a significant influence on the corrosion performance of aluminum alloys [5,11,12], though the chemical properties of an alloying element should be very different from those at its elementary substance state [13].

Microstructure is a crucial factor that influences the corrosion performance of aluminum alloys [14]. Particularly, the amount, size, location, and chemistry of precipitated phases show significant effects on the pitting corrosion behavior of aluminum alloys [8,15,16,17,18]. Abundant studies have shown that the addition of Li can change the microstructures of Al alloys dramatically [2,3,7,19,20,21,22], especially for the precipitated phases induced by Li addition. For instance, the addition of lithium to 0.5 wt.% facilitates the precipitation of θ′ (Al_2_Cu) phase with a refined dispersion in the matrix of 2xxx series aluminum alloys, while higher lithium content (1.0 wt.%) results in the formation of T_1_ (Al_2_CuLi) as the dominant precipitant [23]. T_2_ (Al_6_CuLi_3_) [16] and δ′ (Al_3_Li) [24,25] phases are also common precipitates in Al–Cu–Li alloys. According to Li et al. [16,26,27], the θ′ phase usually acts as the cathode compared to the matrix, while T_1_ and T_2_ are anodic to the alloy base, indicating that further addition of lithium can change the initiation behavior of pitting corrosion. However, the evolution of pitting corrosion on aluminum alloys in response to lithium content has not been systematically studied. Moreover, Carrick et al. [11] reported that the susceptibility of intergranular corrosion and pitting corrosion is higher for lithium-containing AA2099 alloy compared with AA2024, which has no lithium, whereas, based on work of Kumai et al. [24], the addition of lithium does not show deteriorative effects on the pitting resistance of Al alloys. The controversy inspires us to carry out careful analyses to clarify the effect of lithium addition on the corrosion sensitivity of Al alloys.

In this paper, which is the first part of a broader study on the subject, the microstructures of 2029, 2060, and 2098 aluminum alloys, having lithium content ranging from 0 to 1.29 wt.%, were characterized. The corrosion behavior of the three alloys were investigated. The mechanisms for the pitting evolution and formation of severe localized corrosion are discussed. This work will be followed by further analysis using the point defect model in the second part of this series.

## 2. Experimental 

### 2.1. Materials and Microstructure Characterization

The chemical compositions of the aluminum alloys used in this study, which are AA2029-T8 (#S1), AA2060-T8 (#S2), and AA2098-T851 (#S3), are listed in Table 1. For the three aluminum alloys from S1 to S3, the compositional difference is mainly the increasing content of lithium, as the content is shown by weight fraction. Magnesium, manganese, and zinc contents are also slightly different. The microstructures of the Al alloys were characterized using an Olympus optical microscope (Tokyo, Japan) and a TESCAN Vega3 scanning electron microscope (SEM) (Brno, Czech Republic). The microstructures were revealed with an etching solution of NaF (0.5 g), HNO_3_ (1 mL), HCl (2 mL), and H_2_O (97 mL). Transmission electron microscopy (TEM) specimens were twin-jet polished in the electrolyte with 25% HNO_3_ and 75% methyl alcohol at −40 °C. TEM characterizations were performed on a FEI Talos F200X microscope (Hillsboro, OR, USA) with an acceleration voltage of 200 kV.

### 2.2. In-Situ Immersion Test

**Figure 1:**
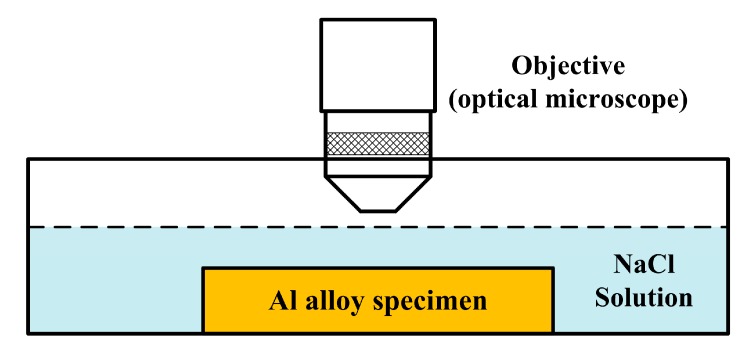
Schematic for in-situ observation of the immersion test.

For the in-situ immersion tests, the experimental set-up is depicted in Figure 1. The specimen was attached to the bottom of a small tank. The specimens were ground to 1200 grit, polished with 1 µm diamond suspension, rinsed with deionized water, degreased in ethanol, and dried with a stream of N_2_ gas. In order to avoid contamination of the objective lens by the solution, a lens with a low magnification (10×) is applied, and the total magnification of the observation system is 50×. Once the solution is poured to the tank, the software records the surface morphology every 1 min. Since pitting corrosion occurs very quickly in Cl^−^-containing solution, we used 0.001 M NaCl solution for the in-situ immersion test, allowing us to effectively record the accumulated numbers of corrosion pits. A higher concentration of Cl^−^ solution (0.6 M NaCl) was chosen for the observation of pitting morphologies, and the 3D morphologies of the pits were characterized by using a Zygo 3D optical surface profilometer (Berwyn, PA, USA).

### 2.3. Electrochemical Measurements

The working electrodes (WEs) with dimensions of 10 × 10 × T mm^3^ were cut from the received specimens, where T is the thickness of the as-received material. The rolling direction is in the 10 × 10 mm^2^ surface, and T is along the short transverse direction. Prior to all experiments, the WEs were ground with SiC papers to 1200 grit, rinsed with deionized water, degreased in ethanol, and dried. The solution for the electrochemical measurements was borate buffer, prepared by 0.0225 M H_3_BO_3_ and 0.11 M Na_2_B_4_O_7_·10H_2_O, with 0.01 M NaCl (pH = 8.4). We used the borate buffer solution for the electrochemical tests in order to keep the pH constant. The solution was deaerated with N_2_ gas, and the measurements were conducted at ambient temperature (20 °C). A Solartron Analytical Modulab (Farnborough, Hampshire, UK) was employed to conduct the polarization measurements. A platinum mesh and a saturated calomel electrode (SCE) were used as the counter electrode and the reference electrode, respectively. A Luggin capillary, which was filled with the same electrolyte as the test solution, was used as a salt bridge. Prior to the electrochemical measurements, the WE was cathodically polarized at −0.5 V (vs. OCP) for 5 min to produce a reproducible surface. Subsequently, the WE was stabilized at the OCP for 900 s. After that, the potentiodynamic polarization curves were recorded with a scanning rate of 0.33 mV/s. Potentiostatic polarization tests were performed by applying −0.45 V_SCE_ to the WEs for 12 h.

## 3. Results

### 3.1. Microstructures

**Figure 2:**
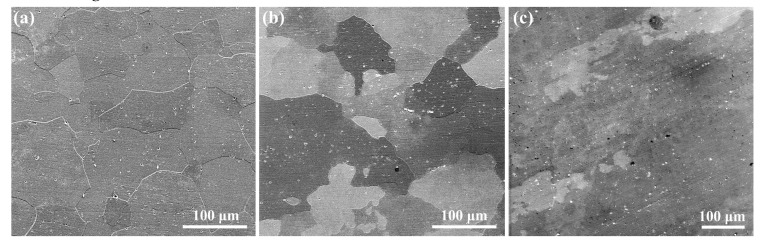
SEM microstructures of the aluminum alloys. (**a**) S1, (**b**) S2 and (**c**) S3.

**Figure 3:**
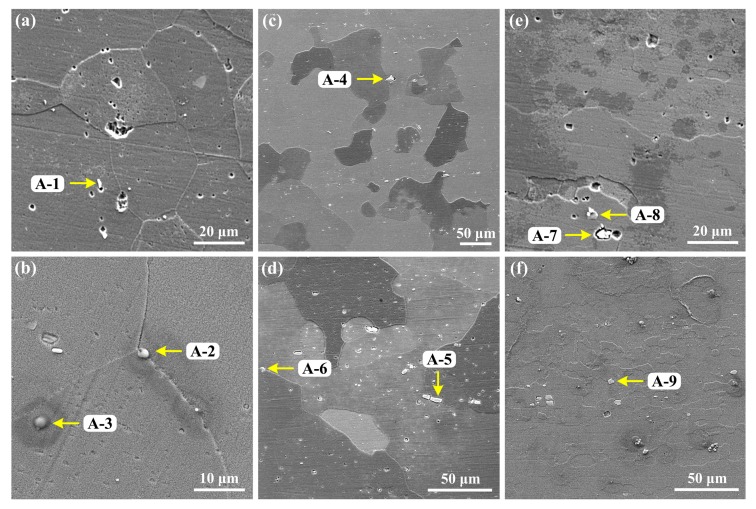
SEM/EDS characterization of intermetallic phases in the aluminum alloys. (**a**) and (**b**): S1, (**c**) and (**d**): S2, (**e**) and (**f**): S3. A-1 to A-9 are the SEM/EDS characterization points.

Figure 2 shows the microstructures of the three types of aluminum alloys. Specimen S1 exhibits equiaxed grain feature with an average grain size of 60–100 μm. In comparison, larger grains decorated with small and irregularly shaped grains can be seen in S2. The microstructure of S3 is distributed along one direction, which should be the rolling direction, as the alloy was stress-relieved by stretching (T851 treatment). Also, it can be seen that S3 shows distinctly different grain morphologies, and very large grains are presented. Many large intermetallic phases are found in the SEM images of the three alloys, energy dispersive spectrum (EDS) characterizations were performed, as shown in Figure 3 and Table 2.

For specimen S1, the large intermetallic particles are mainly located at the grain boundaries, this characteristic is demonstrated by the large pits, caused by etching, at the grain boundaries in Figure 3a. In Figure 3c,d, specimen S2 also shows large intermetallic phases at the grain boundaries, but the size and distribution of particles in specimen S3 seem to be even within and in between the grains. As for the chemical composition, all the intermetallic phases marked by the yellow arrows in Figure 3 primarily contain Al, Cu, and O. However, by comparing the shape of the intermetallic phases, the Cu content, and the ratio of Al:Cu (Al/Cu), we found that the spherical and quasi-spherical particles possess lower Cu content (Al/Cu is around 3), while those with the narrow strip and irregular shapes contains very high Cu content. The relationship between the shape and composition of the intermetallic phase is out of the scope of the present paper and will not be discussed further.

**Figure 4:**
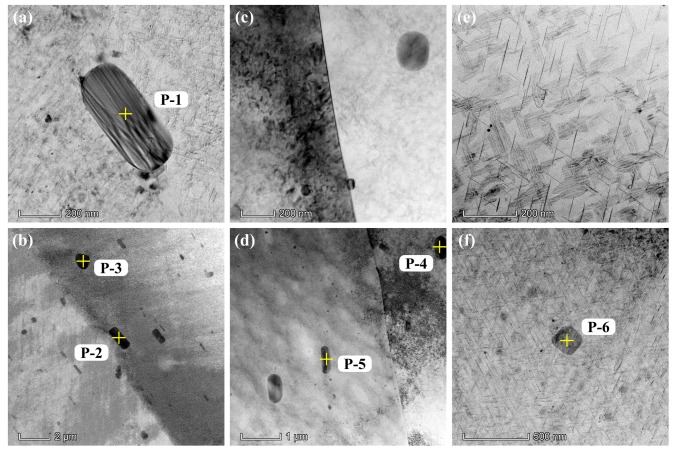
TEM images of the aluminum alloys. (**a**) and (**b**): S1, (**c**) and (**d**): S2, (**e**) and (**f**): S3. P-1 to P-6 are the STEM/EDS characterization points.

The characteristics of the small intermetallic phases were analyzed by TEM, as shown in Figure 4. Scanning transmission electron microscopy (STEM) mode was used to carry out EDS characterizations. A large number of intermetallic phases are found in the three alloys by TEM characterization, but the microstructure of specimen S3 is distinctly different from that of S1 and S2. The needle-shaped precipitates in S3 are a typical feature of the T_1_ phase (Al_2_CuLi) [3,28], whereas larger phases like the size of “P-6” in Figure 4f are rarely seen in S3 by TEM. The chemical compositions of the intermetallic phases marked P-1 to P-6 are listed in Table 3. The intermetallic phases P-1 to P-3 of specimen S1 show the highest Mg content (around 1 wt.%) and are regarded as being the S phase (Al_2_CuMg) [29]. In comparison, the P-4 and P-5 phases in S2 have higher Mn content than the other two alloys, and the Mn content of P-4 is up to 11.96 wt.%, which could correspond to Al_20_Cu_2_Mn_3_ [30]. The P-6 phase in specimen S3 mainly possesses Al and Cu and no Mg, and little Mn was detected. It should be noted that, according to Table 1, specimens S2 and S3 contain Li, but it was not detected due to the limitation of the TEM used for the present characterization. Hence, P-6 could be an Al–Cu or Al–Cu–Li intermetallic phase.

### 3.2. Electrochemical Measurements

**Figure 5:**
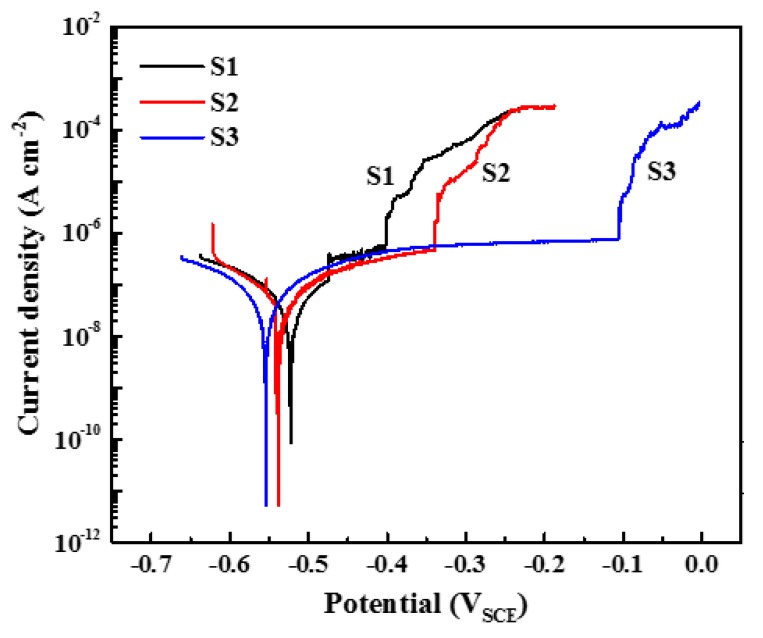
Potentiodynamic polarization curves of aluminum alloys in borate buffer solution with 0.01 M NaCl.

**Figure 6:**
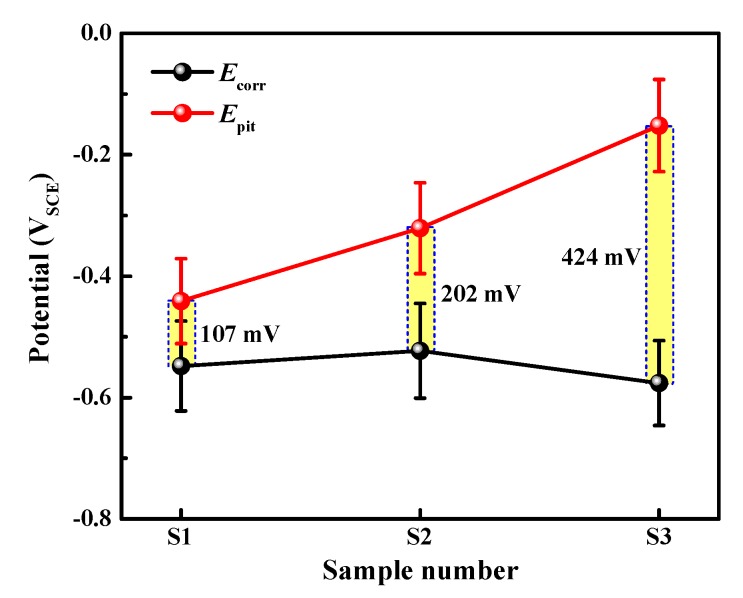
Comparison of the corrosion potentials and pitting potentials of the three types of aluminum alloys. The data was obtained based upon 9 parallel experiments for each specimen.

The corrosion performance of the aluminum alloys was analyzed by potentiodynamic polarization measurement in borate buffer solution with 0.01 M NaCl, and the results are shown in Figure 5. It should be noted that the potentiodynamic test for each specimen was repeated nine times, and the results shown here are the ones that are closest to the average values of corrosion potential (*E*_corr_), pitting potential (*E*_p_), and passive current density (*i*_p_). In Figure 5, the order of *E*_p_ is S1 < S2 < S3, indicating that sequence of pitting resistance is S1 < S2 < S3. However, the passive currents are similar for all three aluminum alloys. The average values of *E*_p_ and *E*_corr_ of the three alloys are depicted in Figure 6. The *E*_pit_ values of S1, S2 and S3 are −441 ± 70 mV, −321 ± 75 mV, and −152 ± 76 mV, respectively, and the *E*_corr_ values of S1, S2 and S3 are −548 + 74 mV, −523 + 78 mV, and −576 + 70 mV, respectively. The gaps between *E*_pit_ and *E*_corr_ are regarded as being the passive range, as marked by the yellow bars. S3 shows the noblest pitting potential and lowest corrosion potential, and, thereby, possesses the widest passive range (424 mV). In comparison, S2 has a slightly larger passive range than S1, indicating the medium pitting resistance of S2. It seems that, for the present lithium content range and corrosion environment, the aluminum alloy with higher lithium content exhibits the better pitting resistance.

**Figure 7:**
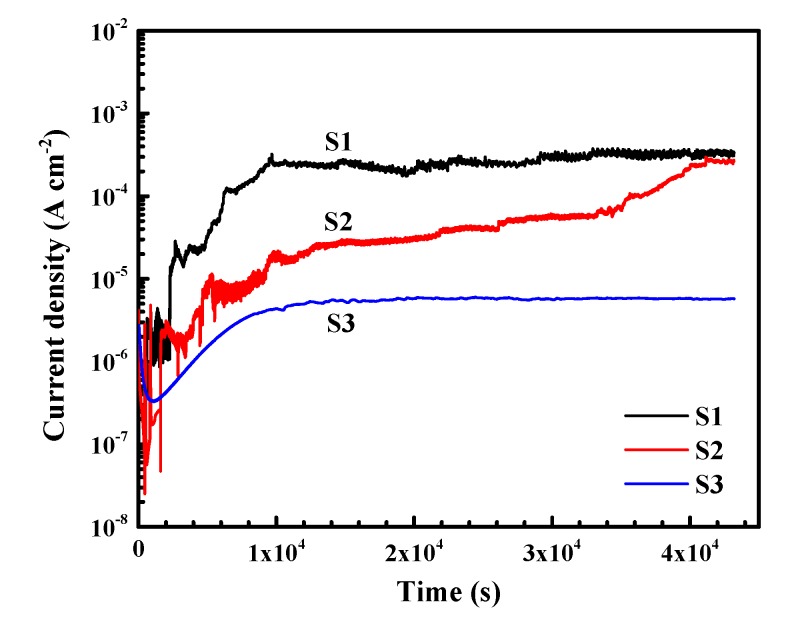
Potentiostatic polarization curves of aluminum alloys in borate buffer solution with 0.01 M NaCl. The applied potential is −0.45 V_SCE_.

The electrochemical corrosion behavior of the aluminum alloys was also analyzed via potentiostatic polarization measurement, and the results are shown in Figure 7. The applied potential (−0.45 V_SCE_) is within the passive region of the alloys. In the beginning period, many current spikes can be found for specimens S1 and S2, which means that the passive films on S1 and S2 are subjected to large numbers of meta-stable breakdown and repassivation events. In comparison, no spike is found on specimen S3, suggesting that the passive film on S3 is more stable. After about 1 × 10^4^ s, the current densities of specimens S1 and S3 reached plateaus, whereas the current density of S2 progressively increases. Finally, the sequence of stable current density (12 h) lies in the order of *i*_S1_ ≈ *i*_S2_ > *i*_S3_. Taking the potentiodynamic results into consideration, it is seen that the addition of lithium could enhance the electrochemical corrosion resistance of the alloys under the current corrosion condition. A low percentage of lithium (0.78 wt.%) shows a moderately positive effect on the resistance to general corrosion, while the addition of 1.29 wt.% lithium improves the pitting resistance significantly.

### 3.3. Immersion Test

#### 3.3.1. Number of Pits

**Figure 8:**
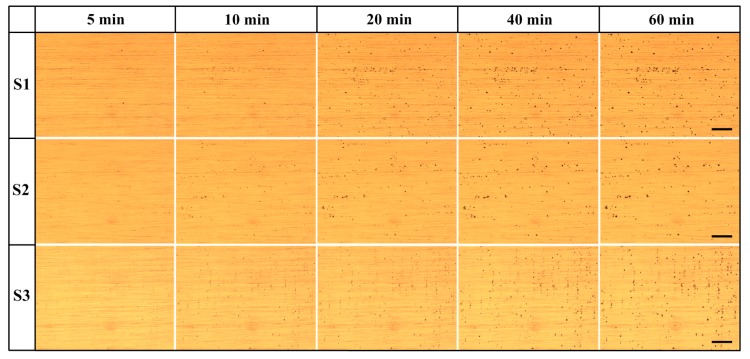
Evolution of pits on aluminum alloys in aerated 0.001 M NaCl solution. Scale bar is 150 μm.

An immersion test is a cogent method for investigating the corrosion performance of metals and alloys. In this study, an in-situ approach is used to observe the development of corrosion on the specimen surfaces. By utilizing the experimental set up depicted in Figure 1, pitting morphologies were obtained, as shown in Figure 8. As pitting corrosion occurs very quickly at higher concentrations of NaCl solution (such as 0.6 M NaCl solution in which pitting occurs within 1 min), we used 0.001 M NaCl solution for the in-situ observation of pitting evolution. Note that the solution used here is without the borate buffer, such that the stable pitting can occur under OCP conditions. In Figure 8, pits can be clearly seen on the three alloys after 5 min of immersion, and more pits are found on the surface of specimen S3 at 5 min than on S1 and S2. With increasing immersion time, more pits appeared on the specimens, and the existing small pits grew to larger ones. Meanwhile, S1 and S2 seem to have larger pits than S3. 

**Figure 9:**
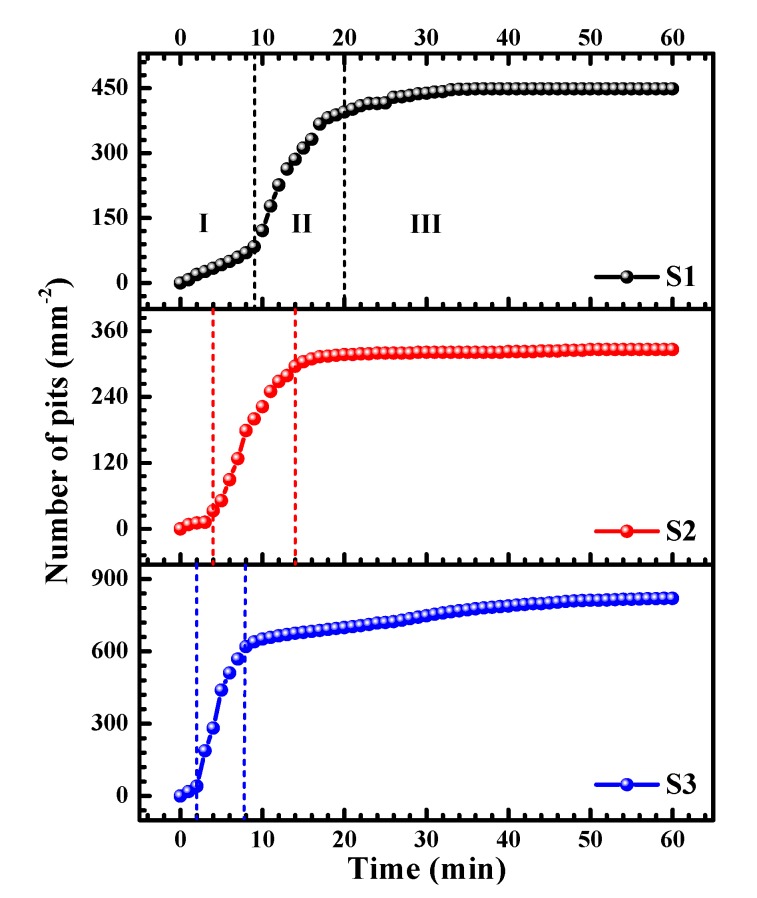
Number of pits vs. immersion time in aerated 0.001 M NaCl aqueous solution.

Figure 9 presents the cumulative numbers of pits (*N*_t_) as a function of immersion time. According to the shape of the curves, the development of pitting events can be divided into three stages: (I) Initial, slow nucleation stage: Pitting begins to occur, but the increasing rate of *N*_t_ is at a low level; (II) Fast development stage: *N*_t_ rises dramatically within a short period; (III) Stabilized growth stage: *N*_t_ increases slowly and finally reach a plateau. The three stages reasonably describe the development of corrosion at the surfaces of aluminum alloys. Pitting corrosion usually occurs at sensitive sites, such as at inclusions, precipitates, grain boundaries, etc. [14]. If these sites are consumed, pitting corrosion is restricted to new positions on the surface. Therefore, the number of pits will gradually reach a plateau (Stage III), although the pre-existing pits may keep growing. In Figure 9, with increasing the lithium content (S1 → S2 → S3), Stages I and II become narrower, indicating that pitting corrosion occurred more quickly. In addition, S3 shows the largest cumulative number of pits (*N*_t_) within Stage III, owing to high lithium content of S3 that contains more pitting sensitive sites. The *N*_t_ of S2 is the smallest, which may be due to the less grain boundaries as a result of coarse grains, leading to fewer pitting sensitive sites, e.g., intermetallic phases. However, it should be noted that fewer pits does not indicate smaller pits or better corrosion resistance. The size of the pits is more critical for the evaluation of corrosion performance, which will be discussed in a later section. 

#### 3.3.2. Morphology of Pits

**Figure 10:**
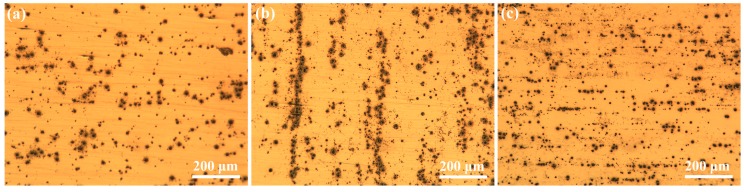
Comparison of the small pits on the aluminum alloys after immersion in aerated 0.6 M NaCl solution for 2 h: (**a**) S1, (**b**) S2, (**c**) S3.

In order to compare the pitting morphologies of the three alloys, a higher concentration of Cl^−^ solution (0.6 M NaCl solution) was used for the immersion test, and the surface morphologies after immersion for 2 h are shown in Figure 10. Specimen S3 has a larger number of pits of smaller size than do S1 and S2. The pits on S2 and S3 are linearly concentrated at localized regions. The linear distribution of pits should be due to the elongated grains induced by rolling processing, suggesting a preferential corrosion feature at grain boundaries such as the T_1_ phase/matrix interfaces for S3. 

**Figure 11:**
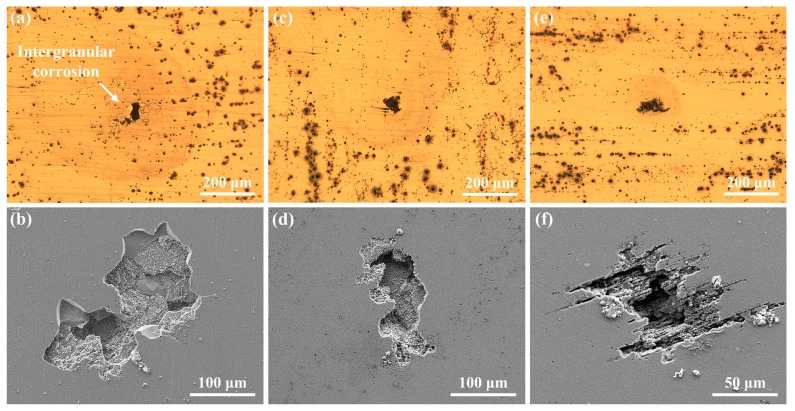
Severe pitting morphologies of the aluminum alloys after immersion in aerated 0.6 M NaCl solution for 2 h: (**a**) and (**b**): S1, (**c**) and (**d**): S2, (**e**) and (**f**): S3. The uppers are optical images, and the lowers are SEM images.

**Figure 12:**
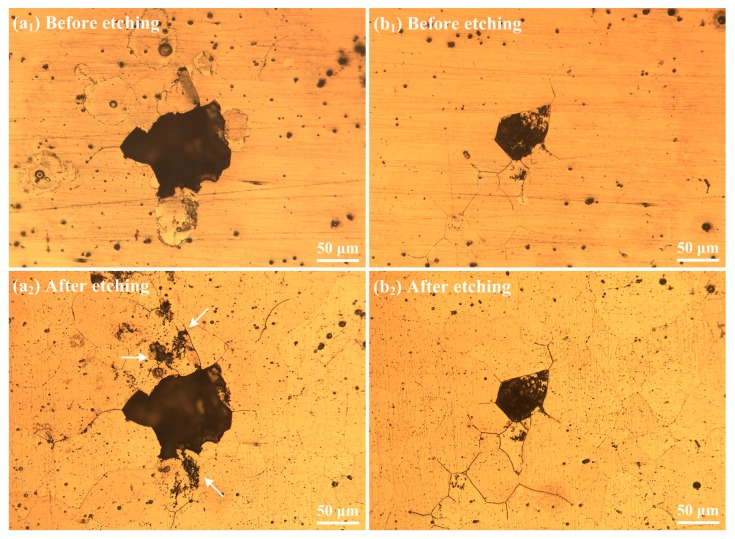
Two representative intergranular corrosion sites on specimen S1 after immersion in aerated 0.6 M NaCl solution for 2 h. The specimens were observed without etching (**a_1_** and **b_1_**) and with etching (**a_2_** and **b_2_**).

Another type of pitting damage, classified as severe localized corrosion (SLC) for aluminum alloys [31], was also found on the alloy surfaces after immersion in 0.6 M NaCl solution for 2 h, as shown in Figure 11. Thus, in Figure 11a, S1 is found to be subjected to intergranular corrosion inside and around the SLC pits, which is evidenced by the grain character inside the SLC pit and shape of the pit edge (see Figure 11b). Detailed examination of the intergranular corrosion features on S1 was performed, and two representative sites are shown in Figure 12. By comparing the morphologies before and after etching, it can be clearly seen that localized corrosion occurred at grain boundaries with an extremely large pit (SLC pit) at the center. According to the shapes of the SLC pits, it is inferred that one or more grains dissolved first at the grain boundaries, i.e., intergranular corrosion occurred to a point that the grain(s) lose bonding with the surrounding matrix and exfoliate from the substrate, leaving an extremely large corrosion pit (50–100 µm opening). The preferred corrosion at grain boundaries for S1 could be due to more intermetallic phases at grain boundaries (see Figure 3a and Figure 4b) in contrast to the other specimens.

#### 3.3.3. Statistical Analysis of Pit Depth

**Figure 13:**
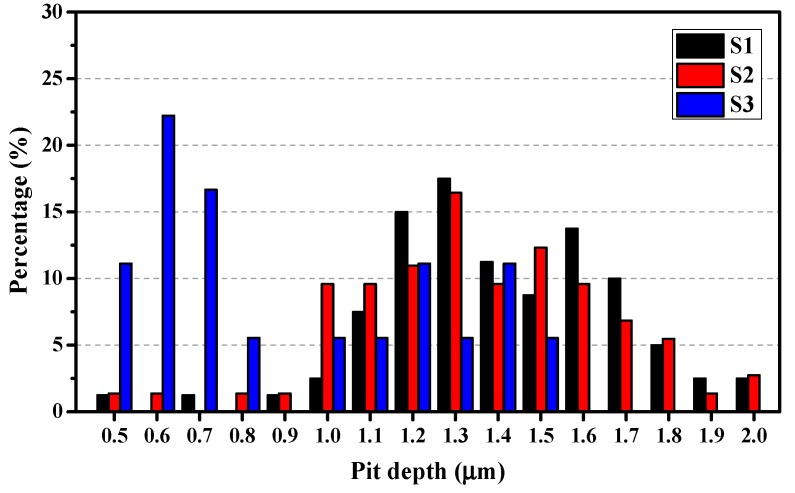
Statistical result for the depth of small pits (depth < 2 μm) after immersion in 0.6 M NaCl for 2 h.

**Figure 14:**
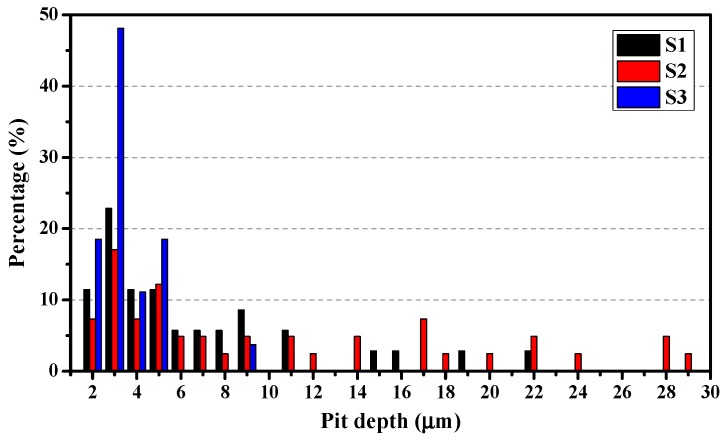
Statistical result for the depth of large pits (depth > 2 μm) after immersion in 0.6 M NaCl for 2 h.

A 3D optical surface profiler was used to conduct a statistical analysis of the pit depth on the specimens. According to the depth distribution of the pits, they are divided into two groups: depth < 2 μm and depth > 2 μm, as shown in Figure 13 and Figure 14, respectively. Note that the 2 μm depth that was used to divide the groups is only for the sake of easier comparison and is of no theoretical significance. Herein, the Percentage (*P*) is calculated via the equation:

(1) $$P\;=\;\frac{n}{N_{t}}\;\times \;100\%$$

where *n* is the number of a specific depth, *N*_t_ is the total number of pits in the present depth range (e.g., in Figure 13, the depth range is 0.5–2 μm). In Figure 13, the distributions of pit depth for specimens S1 and S2 are similar, and their peaks emerge at 1.3 μm. However, the largest *P* for S3 is at 0.6 μm (~22%), indicating that S3 has shallower pits within this range. 

**Figure 15:**
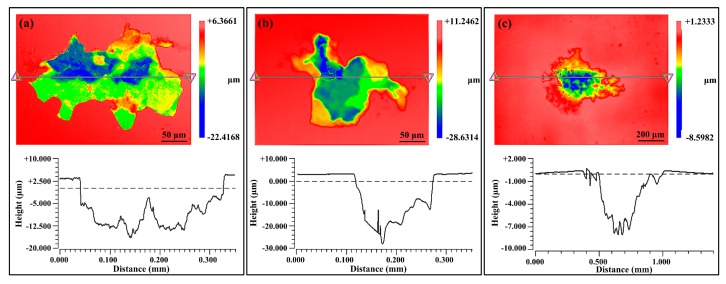
3D depth profile of the deepest pit on each alloy after immersion in 0.6 M NaCl solution for 2 h: (**a**) S1, (**b**) S2, (**c**) S3.

In Figure 14, the peak values of the three alloys appear at 3 μm. Nearly 50% of the pits on S3 have such depth, and the distribution of the pit depth of S3 is roughly between 2–5 μm. In comparison, the pit depths of S1 and S2 are widely distributed, particularly for alloy S2, many deep pits (>20 μm) can be found. Considering the cumulative numbers of pits in Figure 9 as well as the TEM characterization results in Figure 4, and despite the fact that S3 has much larger number of sensitive sites, the small precipitates in S3 have not caused severe corrosion. The profile images of the deepest SLC pits on the three alloys are shown in Figure 15. The depths of the SLC pits on S1, S2, and S3 are 22.4 μm, 28.6 μm, and 8.6 μm, respectively. Clearly, the “mouth” size of the SLC pits may be similar, while the depths of the SLC pits may be very different, which is a testament to the importance of this analysis. 

## 4. Discussions

### 4.1. Effect of Li on Pitting Corrosion

From the polarization results in Figure 5 and Figure 7, it is indicated that the addition of lithium promotes the pitting resistance of Al alloy in borate buffer solution. The alloy with higher lithium content shows wider passive range and more stable passive current, suggesting a better passivation behavior. According to Wang et al. [32], lithium participates in the process of film formation and contributes to lower density of oxygen vacancies in the aluminum oxide film. The lowered density of oxygen vacancies decreases the absorption of aggressive anions, the Cl^−^ ion in this case, into oxygen vacancies and, thereby, retards the vacancy condensate at the metal/film interface, leading to enhanced pitting resistance. This mechanism well supports the electrochemical findings of the present work.

As can be seen from the in-situ observation of the pitting processes of S1, S2, and S3 in 0.001 M NaCl solution (Figure 8 and Figure 9), the development of pitting corrosion can be divided into an initial, slow nucleation stage (Stage I), a fast development stage (Stage II), and a stabilized growth stage (Stage III). The difference in the development of pitting for the three alloys is the duration of each of the stages. With increasing lithium content, the durations of Stages I and II become shorter. Combining this fact with the SEM and TEM microstructural analyses, the higher rate of pitting initiation and development should be attributed to the higher activity of lithium and the larger numbers of lithium-containing intermetallic phases, such as the abundant T_1_ precipitates in S3 (see Figure 4e,f) [33,34,35]. Electrochemical corrosion would take place due to the microcell reaction between the secondary phases and the matrix. The mechanism for the microcell reaction has been well established, based upon the chemical composition of the precipitates, i.e., with/without lithium for Al–Cu–Li alloys, which decides if the precipitate acts as the anode or the cathode within the cell [16,26,27]. 

In addition, the cumulative number of pits lies in the order of S3 > S1 > S2 (see Figure 9), while the sequence of pit depth is S3 < S1 ≈ S2 (Figure 13 and Figure 14). This could be due to the greater amount, but smaller size, of the T_1_ phases in S3, which act as abundant but small anodes, leading to larger numbers of small pits. In comparison, S1 and S2 have fewer, but much larger, precipitates, such as the S phase for S1 and Al_20_Cu_2_Mn_3_ phase for S2, respectively, resulting in large pits, but in smaller numbers.

### 4.2. From Pitting Corrosion to SLC Pits

**Figure 16:**
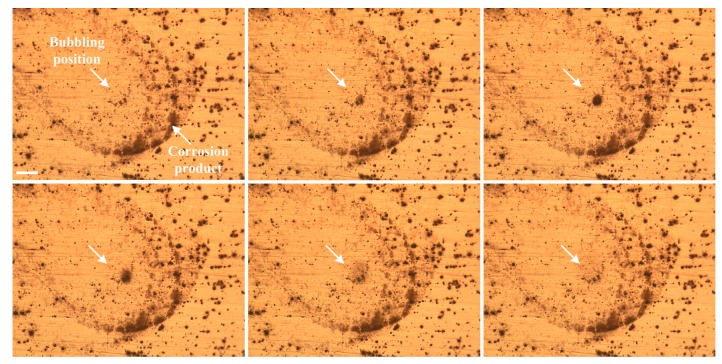
In-situ observation of a gas bubbling site on specimen S1 in aerated 0.6 M NaCl. Scale bar is 150 μm.

In Figure 11, circular features are observed around the SLC pits. The generation of SLC pits and circular features can be interpreted based upon the in-situ observation results of gas bubbling from the site on S1 after immersion for 105 min, as shown by the video captured in Figure 16. A similar feature has been described by Bargeron and Givens [36,37]. In Figure 16, as marked by the arrows, a gas bubble was generated in a pit at the center of the circle, then left the alloy surface and finally broke. This process continued at this site during the immersion test, resulting in the continuous consumption of material inside the pit and leading to the formation of a SLC pit. The morphology of the pit, after cleaning the corrosion product, is exhibited in Figure 11a. The gas inside the bubble is presumed to be hydrogen, due to the autocatalytic acidification effect at the tip of SLC pit, which may involve the following reactions for S1 [30]:

(2) $$H_{2}{O\;=\;H}^{+}{\;+\;\mathrm{OH}}^{-}$$

(3) $${\mathrm{Al}\;=\;\mathrm{Al}}^{3+}{\;+\;3e}^{-}$$

(4) $${\mathrm{Al}}^{3+}{\;+\;3H}_{2}O^{-}{\;=\;\mathrm{Al}(\mathrm{OH})}_{3}{\;+\;3H}^{+}$$

According to Reaction (4), $H^{+}$ is produced by the hydrolysis of ${\mathrm{Al}}^{3+}$ to form ${\mathrm{Al}(\mathrm{OH})}_{3}$, thereby lowering the pH at the pit tip. Reaction (3) provides the electrons that will react with H^+^ to form H_2_ via the following Reaction (5).

(5) $${2H}^{+}{\;+\;2e}^{-}{\;=\;H}_{2}↑$$

However, it is to be noted that Bargeron and Givens [36,37] observed similar gas evolution, but the gas was identified to be H_2_S, NH_3_ or CH_4_, depending upon the identity of the pit nucleation sites (presumably, sulfide, nitride, and carbide inclusions, respectively).

Reactions (2)–(5) explain the mechanism for the bubbling that continuously occurs at the pitting sites and lead to SLC. The circular feature around the bubbling sites may be attributed to incident spherical shock waves in the liquid upon the collapse of the bubbles [38], pushing away products from the re-precipitation of Al^3+^ as Al(OH)_3_ from the solution onto the alloy surface external to the pit via Reaction (4). Interestingly, although the corrosion products had been removed, the circular feature can still be clearly seen in Figure 11, possibly because of the occurrence of under-deposit corrosion.

**Figure 17:**
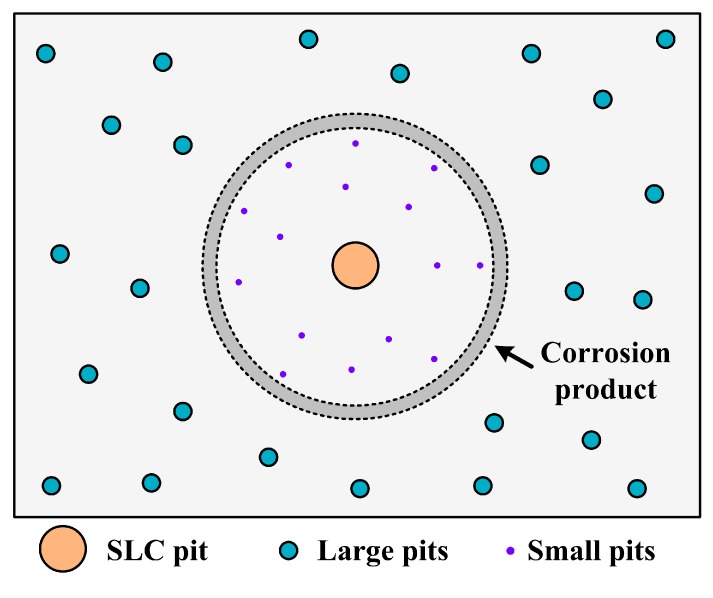
A schematic for the distribution of various sizes of corrosion pits at and near a bubbling site.

In addition, it can be seen from Figure 11 that the sizes of the SLC pits are much larger than the surrounding pits, whereas the pits which are adjacent to these SLC pits are smaller in size and fewer in number than those that are further away from the SLC pits. This characteristic is schematically depicted by Figure 17, which is explained by the formation of local microcells: the anodes are the SLC pits, and the adjacent regions are cathodically protected to some significant extent, thereby reducing the nucleation and growth rates of the surrounding pits. Similar morphologies were reported by Donatus et al. [30] for 2198 Al alloy and Ma et al. [31] for 2099 Al alloy. This phenomenon has been previously explained in terms of a “hemisphere of influence (HOI)” centered on the well-established, central pit [39]. The HOI is established by the consumption of the coupling current on from the pit on the external surface by hydrogen evolution and/or oxygen reduction, resulting in a local increase in pH and hence in the precipitation of Al(OH)_3_. As the pit ages, it demands more resources from the external surface in terms of the cathodic reaction, resulting in an increase in the throwing power of the current from the pit and hence in the radius of the HOI.

To understand the mechanism for the very few SLC pits on specimen S3 which possesses the highest Li content, the effect of Li on the acidic level inside the tip of the pits should be considered. According to Reactions (2) to (5), the low pH environment at the tip of the pits is critical for the formation of SLC pits. As Li is very active metal, in the acidic environment at the tip of a pit, the following Reaction (6) may occur along with Reaction (3) [26]:

(6) $${\mathrm{Li}\;=\;\mathrm{Li}}^{+\;}{\;+\;e}^{-}$$

Hence, the H^+^ inside the pit will be consumed by the electrons via Reaction (5), and the pH of the pit is lowered. In other words, lithium could play a critical role in neutralizing the environment and inhibiting the growth of pits into the alloy. Such an effect would be very beneficial for specimen S3, as can be seen from the results of electrochemical measurements (Figure 5 to Figure 7) and immersion tests (Figure 8 to Figure 15). However, the advantageous effect of lithium is overwhelmed by the deteriorative effect of the large intermetallic phases that result in the severe corrosion on specimen S2. 

## 5. Conclusions

The microstructures and pitting corrosion behavior of AA2029-T8 (S1), AA2060-T8 (S2), and AA2098-T851 (S3), with increasing lithium content, were studied using SEM, TEM, EDS, electrochemical measurements, in-situ observation, and statistical analysis of pit depth observed in immersion test. The principal conclusions are as follows:

(1) The pitting potential of the three alloys lies in the following order: S1 < S2 < S3. With the comparatively highest lithium content, S3 shows the widest passive region and lowest steady-state current density.
(2) The development of the pitting process can be divided in to an initial slow nucleation stage (Stage I), a fast development stage (Stage II), and a stabilized growth stage (Stage III). The accumulative number of pitting sites rises more quickly with increasing lithium content (S1 → S2 → S3), whereas S1 and S2 possess deeper pits than does S3.
(3) Circular characteristics were found near the SLC sites, which should be due to the precipitation of corrosion products on the alloy surfaces external to the pit.
(4) A well-established pit partially protects the surrounding area cathodically, thereby inhibiting the nucleation and growth rates of new pits under the “hemisphere of influence” of the central pit.

## Figures and Tables

**Table 1:** Chemical compositions of the three aluminum alloys (wt.%).

Number	Material	Cu	Li	Mg	Mn	Ag	Zr	Zn	Al
S1	2029-T8	3.46	-	0.80	0.26	0.04	-	0.01	Bal.
S2	2060-T8	3.63	0.78	0.67	0.25	0.04	0.06	0.29	Bal.
S3	2098-T851	3.71	1.29	0.26	0.03	0.03	0.06	0.01	Bal.

**Table 2:** SEM/EDS analyzing results of points A-1 to A-9, as marked in Figure 3. (wt.%).

Element		Al	Cu	O	Al/Cu
**S1**	A-1	13.01	69.94	17.05	0.186
	A-2	43.75	13.28	42.97	3.294
	A-3	35.68	12.09	52.23	2.951
**S2**	A-4	18.85	72.84	8.31	0.259
	A-5	15.59	69.43	14.98	0.225
	A-6	49.30	42.66	8.04	1.156
**S3**	A-7	2.13	87.92	9.95	0.024
	A-8	56.74	17.06	26.20	3.326
	A-9	19.74	71.51	8.75	0.276

**Table 3:** STEM/EDS analyzing results of points P-1 to P-6, as marked in Figure 4. (wt.%).

Element		Al	Cu	O	Mg	Mn	C
**S1**	P-1	90.76	6.03	1.99	1.22	-	-
	P-2	92.67	5.00	0.19	1.18	0.96	-
	P-3	85.39	7.59	0.21	0.99	5.82	-
**S2**	P-4	75.72	10.98	-	-	11.96	1.34
	P-5	74.26	9.55	-	0.68	9.77	5.74
**S3**	P-6	87.21	11.20	0.83	-	0.76	-
